# What influences universities’ regional engagement? A multi-stakeholder perspective applying a Q-methodological approach

**DOI:** 10.1080/21681376.2019.1578258

**Published:** 2019-05-08

**Authors:** Verena Radinger-Peer

**Affiliations:** Department of Economics and Social Sciences, Institute for Sustainable Economic Development, University of Natural Resources and Life Sciences Vienna (BOKU), Vienna, Austria.

**Keywords:** university engagement, Q-methodology, organizational-field factors, organizational-internal factors, viewpoints

## Abstract

This paper argues that rather than being a process that can be objectively planned or predicted, regional engagement on the part of universities is a learning activity featuring characteristics of a subjective deliberation process. This subjective deliberation process is simultaneously influenced by factors operating at the intra-organizational and regional level, as well as the field in which the university is located. A model that takes this multilevel environment into account is applied to a single case study region: Kaiserslautern (Germany). A Q-methodological approach is employed to reveal and analyze the aforementioned subjective perspectives regarding drivers of universities’ regional engagement. Two key viewpoints emerge from this analysis: one perspective reflects a highly institutionalized reading of regional engagement, and can be traced to the strong policy push to create universities as drivers of regional development in the Palatinate. The second perspective recognizes greater diversity among the various engagement activities pursued by universities beyond this narrow institutional engagement, driven through individual interaction with regional partners. These insights question the widespread pipeline-dominated perspective on universities’ regional engagement and argue for a more systemic understanding of the role of higher education institutions within their region.

## INTRODUCTION: UNIVERSITIES’ REGIONAL ENGAGEMENT AS A MULTIFACETED PHENOMENON

While it is commonly accepted that universities are key repositories of new knowledge and highly qualified graduates, the roles they are expected to play have changed significantly over the past 30 years. It was in the 1980s when claims regarding universities’ regional engagement first arose in regional policy. In contrast to the first two pillars – research and teaching – regional engagement is a multifaceted and hard-to-demarcate phenomenon. Various perspectives exist on the mode of interaction with the surroundings (the how) as well as the types of activities pursued (the what). These perspectives reveal that universities’ interactions with their respective region have a dual nature. This duality refers, on the one hand, to contributions via linear, direct knowledge transfer activities (pipeline-dominated perspective), while, on the other, it entails contributions that emerge from formal and informal participation in regional networks (Boucher, Conway, & Van der Meer, 2003), collective action and knowledge co-production with various actors from multiple backgrounds. Apart from differing perspectives on how universities’ engage with ‘their’ region, their specific activities also vary content-wise. A general differentiation is between commercialization-based activities (e.g., patenting and licensing of innovations, academic entrepreneurship) (Perkmann, Tartari, McKelvey, & Autio, 2013) and non-commercialization-based activities (Lassnigg, Trippl, Sinozic, & Auer, 2012). The latter include capacity-building and steering activities that contribute to regional social, cultural and environmental needs (Boucher et al., 2003; Goddard & Vallance, 2011). In the present paper, regional engagement is defined as interactions between the university and its location region via teaching, research and other knowledge-transfer activities which (1) have a regional scope, (2) tackle regional problems or (3) generate positive impacts for selected regional stakeholders/the region as such. University engagement involves person-to-person interactions that link universities and other organizations (Cohen, Nelson, & Walsh, 2002). As such, university engagement is not merely a one-sided process but depends also on the regional actors’ willingness and capacity to cooperate (Peer & Penker, 2014).

The multifaceted nature of universities’ regional engagement is the reason for the prevailing lack of understanding about why and how specific types of regional engagement activities occur or do not occur in certain university–region settings. So far investigations into the commercialization-based activities of universities, their incidence, likelihood and impact dominate the scientific discourse (e.g., Breznitz & Feldman, 2012; D’Este & Patel, 2007; Perkmann et al., 2013). Factors such as age, scientific field, position, gender, industry characteristics and others have been identified as influential regarding regional innovation performance.

These scientific findings have nurtured the political conviction that universities’ regional engagement is strategically ‘plannable’. This is reflected in the institutionalization of technology transfer in the form of technology transfer offices (liaison offices, patent offices), the standardization of the attendant processes, as well as the adaptation of regulative frameworks in order to reward these kinds of activities. However, this pipeline-dominated approach covers only a small part of universities’ regional engagement activities and provides an incomplete representation of the wider processes of knowledge exchange and co-production, networking and collaboration between university members and regional stakeholders (Abreu, Grinevich, Hughes, & Kitson, 2009; Ulrichsen, 2014).

Various indications in the literature (see also below) lead the author to question the pipeline-dominant perspective and, consequently, the assumed potential to strategically ‘plan’ universities’ regional engagement activities. First, universities are complex organizations of loosely coupled entities (Weick, 1976) with high autonomy for the individual researchers (Pasternack, Sebastian Schneider, & Zierold, 2015). Furthermore, engagement is just one of the multiple agendas, activities and incentives influencing universities and university academics, and the regional level is merely one of multiple scales at which the university is active (Benneworth, Pinheiro, & Karlsen, 2017). As a consequence, the author argues that regional engagement activities are the result of a subjective deliberation process shaped by the multilevel environment in which the university is situated.

The contribution of the present paper is to reflect on the interplay between these three factors, namely (1) the multilevel nature of the model of action displayed by individual university members, (2) the high autonomy of the individual within academia and (3) the low degree of institutionalization of regional engagement (especially with regard to non-commercial activities). The paper addresses this by asking the research question: Which organizational, regional and organizational-field-level factors most strongly influence the individual deliberation process related to regional engagement? To answer this question, the paper develops a conceptual model that aims to grasp the factors from the multilevel environment which influence this subjective deliberative process. This is applied to a single case study region, Kaiserslautern (Germany), where a Q-methodological approach involving 18 key stakeholders is applied. Q-methodology offers the opportunity to highlight subjective perspectives on commonly occurring themes. These individual perspectives are clustered through factor analysis to reveal shared viewpoints on the topic. Actors from university management and faculty as well as regional actors from politics, business and regional development are involved to capture various perspectives on the occurrence of and drivers behind universities’ regional engagement. By identifying a number of key factors influential in these deliberations other than those typically highlighted in the literature, this paper argues for a more systematic consideration of these subjective deliberation processes to improve understanding of university engagement.

## LITERATURE REVIEW AND MULTILEVEL MODEL

The literature review depicts the current scientific discourse on factors influencing the occurrence of universities’ regional engagement activities and derives a multilevel model that leads the further operationalization and analysis.

### Internal and contextual factors influencing university–regional engagement

Higher education institutions (HEIs) have been subject to various reforms during the last decades. These reforms aim to modernize the higher education (HE) sector in order to increase its efficiency and effectiveness, and to orient its service more towards the expectations of the customers (Broucker & DeWit, 2015). These reforms, which have also taken place in other areas of the public sector, are manifestations of new public management (NPM). Characteristics of NPM include, among others, increased competition for government-provided resources, growth in student fee charging, autonomy to set strategic goals, performance measurement and evaluation, as well as new management styles.

These developments, which have brought greater autonomy to each organization, lead at the same time to increased pressure to demonstrate the organizations’ legitimacy. This balancing act between university autonomy and institutional survival in a competitive environment shapes universities’ strategic deliberations. Within this strategic deliberation process universities try to anticipate what they must do to perform effectively and legitimize their existence (Broucker & DeWit, 2015) and further come up with concrete actions, policies and investments. In doing so, they seek to respond to a set of external factors operating at multiple levels, while they are at the same time dependent on intra-organizational and individual-level determinants. Institutional theory, especially the open-system perspective, emphasizes the influence of the environment on an organization. Modern universities may be referred to as open systems, where complex and dynamic reactions between the organization and its environment mediate through its structural operations, and hence are indeed sensitive to these influences from the external environment (Scott, 2001). Hence, institutional theory provides a helpful basis from which to conceptualize this multilevel environment:

- *Individual level determinants* of regional engagement are relatively well explored and are ascribed an important role in predicting academic engagement (Perkmann et al., 2013). Among the most influential factors are gender, academic age, experience and group-level norms. Thus, male academics (Link, Siegel, & Bozeman, 2007) with high seniority (Boardman, 2009; D’Este & Perkmann, 2011) who are successful in acquiring government grants and funds are most likely to engage in university–region collaborations. Given that engagement is often seeded by personal contacts, more experienced researchers are furthermore likely to have larger networks and hence more social capital (Giuliani, Morrison, Pietrobelli, & Rabellotti, 2010). The existence of mutual trust and commitment (European Commission, 2011), shared goals and previous collaboration experiences (Bishop, D’Este, & Neely, 2011) additionally spur regional engagement. Louis, Blumenthal, Gluck, and Stoto (1989) find that individual behaviour is strongly moderated by the effect of group-level norms. Perkmann et al. (2013) see no conclusive evidence on the role of formal organizational support structures or peer effects for stimulating academic engagement.
- *Organizational level determinants*: Goddard and Vallance (2011), among others, emphasize the type of university and its founding idea as influential factors, and underpin these claims by the example of the Land Grant University in the United States as well as the civic university in the UK (Goddard & Vallance, 2011). The age of the institution is often related to these factors. Boucher et al. (2003) state that younger institutions are often built with a limited tradition of research but with a specific regional mission. A further potentially relevant factor is the balance between applied and basic research. Abreu et al. (2009) conclude, based on a comprehensive survey of UK academics, that the distinction between basic and applied research is overemphasized, although those disciplines with the highest degree of basic research (e.g., physics, mathematics, humanities) turn out to be the least engaged within their region.
- *Regional level determinants*: with regard to the regional environment, perceptions of universities’ roles within a coherent regional development strategy (Kitagawa, 2004) as well as their participation in regional networks and governance structures (Pinheiro, 2011) influence their motivation towards engagement. In relation to this the absorptive capacity of a region – defined as the amount of human capital, culture of and experience with university–region interactions as well as geographical proximity (D’Este & Perkmann, 2011; Trippl, Sinozic, & Lawton Smith, 2012) – is highlighted as essential. Furthermore, regional engagement activities are moderated by the number of universities and other HEIs within the region as well as the degree of competition and/or collaboration between them (Uyarra, 2010).
- *System-level determinants*: Conway, Humphrey, Benneworth, Charles, and Younger (2009) highlight the importance of the institutional approach of a country, specifically direction of the HE system towards engagement. Despite this, research on academic engagement has rarely addressed the role of institutional environments and national policies, partly because engagement has enjoyed less attention from policy-makers than commercialization (Perkmann et al., 2013).

There has to date been no systematic consideration of the emergent ways in which these various determinants come together to create real effects on institutions and the decisions by individual university members to become regionally engaged. It is hence of interest how the simultaneous influence of the aforementioned multilevel environment is perceived and how intra-organizational and external determinants rank in importance. Not all these influencing factors are in control of the university management. This raises the question of how university management can set up strategies for regional engagement given diverse factors and manifold pressures which are beyond their influence.

Different driving forces emerge from this multilevel environment which influence the subjective deliberation process around regional engagement. The nature of these driving forces may be regulative, normative or cultural–cognitive (Scott, 2001). For the HE sector regulative forces entail laws (HE legislation) and regulations (strategies, funding programmes) affecting the organizational structure and operations in a binding, coercive manner. Normative forces include values and norms. In the HE sector these refer to the goals and objectives which are set up within the HE system as a whole, but also within the individual institution in conjunction with the appropriate means to pursue them (e.g., mission statement, financial alignment). Within the university norms and values can also be influenced by interest groups and may affect how an individual perceives their appropriate ‘role’ in a specified social position (e.g., expectations towards the role as rector, dean, group leader or scientist). The cultural–cognitive influence refers to routines and standards which have been established as ‘the way we do things’ with a certain degree of taken-for-grantedness. Within the HE sector this may refer to routines regarding communication and interdisciplinary collaboration within the university, as well as routines and communication structures with regional partners etc. The cultural–cognitive nature of these influencing factors is based on common beliefs and shared logics, enforcing mimetic mechanisms.

### Towards a multilevel model for understanding university regional engagement influences

The various factors from the multilevel environment that are expected to influence individual employees’ actions are conceptualized in a multilevel model which differentiates between three levels (Table 1) (see also Pinheiro, 2011).

**Table 1.** Multilevel model of the factors influencing universities’ regional engagement.

Level	Influencing factors
Organizational level (U)	The founding history as well as reasons for founding the university (‘historical imprinting’) The self-perception of the university The university’s internal incentive system Individual personalities (‘frontrunners’, ‘institutional entrepreneurs’) Need for third-party funds
Regional level (R)	The regional absorptive capacity and willingness to cooperate Economic policy and regional policy and their programmes and strategies Expectations towards regional engagement that are published in the regional and national media Regional networks and governance structures
Organizational-field level (OF)	Other higher education institutions (HEIs) and the collaboration or concurrence with them as well as their role model effect The national and international higher education system Higher education legislation Higher education policy and research policy of the national government Evaluation, prizes and awards by the public sector Public subsidies and funding programmes

The first set of influencing factors are those that arise within the university itself, and five components are distinguished here: (1) the founding history as well as reasons for founding the university, or ‘historical imprinting’ (Scott, 2001); (2) the self-perception of the university (written in mission statements, development plans, etc.); (3) the university’s internal incentive system (e.g., awards, teaching obligations, achievement recognition); (4) individual personalities including ‘frontrunners’ and ‘institutional entrepreneurs’ (Radinger-Peer & Pflitsch, 2017) who act as role models; and (5) the need to attract third-party funds.

There are two components to the geographical environment of the host region: territorial and relational. The territorial element is defined by the spatial domain designed by the administrative or geographical barriers of the territory within which the HEI is located. The relational component implies regional innovation system (RIS) network structures, partnerships and social capital which define the regional boundaries. The factors within the host region which may exert influence on regional engagement activities comprise: (1) the regional absorptive capacity and willingness to cooperate; (2) economic policy and regional policy and their programmes and strategies; (3) expectations towards regional engagement which are published in the regional and national media; and (4) regional networks and governance structures.

The organizational field conceptualizes the interactions between diverse, interdependent organizations that participate in a common meaning system (DiMaggio & Powell, 1983). For universities the following elements are part of the organizational field: (1) other HEIs and the collaboration or concurrence with them, as well as their role model effect (Pinheiro, 2011); (2) the national and international HE system; (3) HE legislation; (4) HE policy and research policy of the national government (Scott, 2001); (5) evaluation, prizes and awards by the public sector; and (6) public subsidies and funding programmes (Table 1).

## THE Q-METHODOLOGICAL APPROACH AND OVERVIEW OF THE KAISERSLAUTERN CASE STUDY

The aforementioned theoretical model is used to answer the research question: Which organizational, regional and organizational-field-level factors most strongly influence the individual deliberation process related to regional engagement? The question is investigated within a single case study, namely, the region of Kaiserslautern, Germany.

### The Q-methodological approach

In the present study it is of foremost interest to reveal the influence of organizational, regional and organizational-field-level factors on the deliberative process towards regional engagement. The overall aim of the Q-methodological study is to identify the dominating viewpoints on this topic among the selected stakeholders.

This work was undertaken within the framework of the FWF project HERO, which is a comparative case study of universities’ regional engagement in three European regions, Kaiserslautern (Germany), Enschede (the Netherlands) and Linz (Austria). The current paper presents results from the Kaiserslautern case study.

The first step was identifying the key stakeholders in this domain from within the university, from the region and also from the wider system-level domain. Apart from scientists, also representatives from the management as well as intermediaries (e.g., technology transfer officers – TTO) from the Technical University of Kaiserslautern and the University of Applied Sciences Kaiserslautern were asked to conduct the Q-sorting exercise. In addition, also cooperation partners from politics (local and regional level), regional development agencies and interest groups representing the local and regional economy were addressed. They were asked to give their subjective perceptions as to the relative importance of various factors in influencing university–region cooperation. Each participant underwent a semi-structured in-depth interview in addition to participating in the Q-methodological ranking procedure.

Q-methodology was developed by William Stephenson as a means of gaining access to subjective viewpoints (Watts & Stenner, 2005). In the study of ‘subjectivity’, Q-methodology combines qualitative and quantitative methods. It is applied to a broad range of topics, including psychological research, political opinion research, market research, environmental psychology research (Müller & Kals, 2004) and agricultural research (Walder & Kantelhardt, 2018), wherever complex subjective structures such as opinions, attitudes and values are examined. The overall aim of the Q-methodological approach is to identify and characterize the dominating viewpoints among the selected stakeholders.

#### Design of Q-instrument and selection of participants

The central interest of the present study was to find out how important the identified factors (Table 1) are to trigger university members to participate in regional engagement activities. Hence, the set of Q-statements (also called the ‘Q-sample’) was formulated based on those factors listed above, as identified from the literature. Statements may consist of terms as well as short sentences. The number of statements depends on the underlying question, though they should aim towards covering all facets of the topic of interest. In the present study the underlying question was: How important are the following factors to trigger university members to participate in regional engagement activities? The statements were derived from a comprehensive literature review and from a pilot case study, based on theoretical assumptions. In the present case they result from the operationalization of the elaborated model (see the second section). The statements consist of influencing factors from the organizational, regional, and organizational field level.

#### Q-sorting procedure

**Figure 1.**
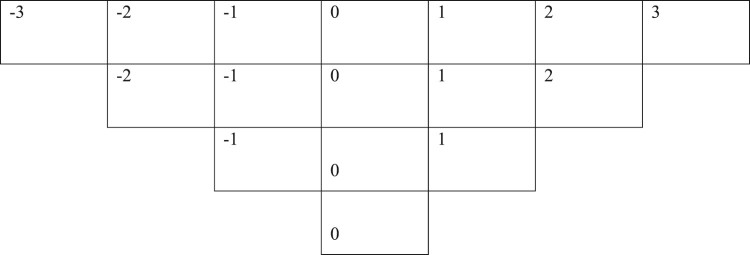
Q-grid ranging from –3 (not important) to 3 (extremely important).

The collected statements are prepared on paper cards. The interview partners are asked to conduct the Q-sort according to a subjective dimension ranging from ‘Is important from my perspective’ to ‘Is not at all important from my perspective’ on a predefined matrix (Figure 1). The idea is that all the statements have to be allocated a ranking within this distribution. First the Q-statements are allocated to three categories of relative importance, namely, important, not important and neutral. In a next step every interview partner sorts the items in a more fine-grained way using a scale ranging from –3, least important, through 0 neutral to 3 extremely important. The interview partners are then allowed to rearrange the statements until they are satisfied with the result. Their comments during the process of sorting the statements provided insightful background information that further support the interpretation of the results. The different sorting patterns (the viewpoints of different individuals) are subsequently compared and contrasted through factor analysis. This process helps to identify groups of participants who make sense of a pool of items in comparable ways, so-called ‘shared viewpoints’ or ‘factors’.

The data gathered via the Q-sorting procedure with 18 interview partners go through three methodological transitions which are unique to the Q-methodology. The first transition is from Q-sorts to factors, via the correlation and factor analysis of the Q-sorts (Table 1); the second is from factors to factor arrays (shared viewpoints), via the weighted averaging of significantly loading or factor-exemplifying Q-sorts (Table 2); the third is from factor arrays to factor interpretations, via the process of interpretation (Watts & Stenner, 2012). A total of 16 Q-sorts were inter-correlated and factor-analyzed using the dedicated computer package PQMethod (Schmolck, 2002).

**Table 2.** Factor characteristics.

Stakeholders	Description^b^	Factor 1	Factor 2
1	R1	0.5558	–0.0927
2	P1	0.5613	0.4280
3	HSW1	0.3107	0.4803
4	HSW2	0.7206	0.0877
5	HSW3	–0.2248	0.7593
6	R2	0.7385	0.1594
7	HSI1	–0.0205	0.5337
8	HSW4	0.6151	0.1087
9	W1	0.3907	–0.0531
10	HSV1	0.7466	0.3677
11	HSI2	0.2063	0.7049
12	HSW5	0.3356	0.2033
13	HSW6	0.0156	0.2097
14	HSW7	0.3932	0.5368
15	W2	0.6952	0.1076
16	HSI3	0.7147	0.4623
17	HSI4	0.2189	0.6814
18	P2	0.1562	0.6034
Number of defining variables	6	4	
Explained variance (%)	24%	19	
Correlation between factor scores	0.2674		

Note: In order to be significant at the *p* < 0.01 level, the factor loading within this study needed to be ≥ 0.608.

The correlation of the Q-sorts reflects the nature and extent of the relationship that pertains between all the Q-sorts in the group. The correlation matrix provides first patterns of similarity and difference from which the factors/shared viewpoints emerge.

As a next step the data-reduction technique of factor analysis is conducted to reveal the key viewpoints. The process of factor analysis involves a statistical inspection of the correlation matrix and attempts to identify distinct regularities or patterns of similarity in the Q-sort configuration generated, and hence patterns in the viewpoints expressed by the participants. A shared viewpoint consists of all those Q-sorts that load significantly on this particular viewpoint and that viewpoint alone. To be significant in the present study at the *p* < 0.01 level, a factor loading needed to be ≥ 0.608 (Table 1).^1^ This criterion can now be applied as a means of identifying these closely approximating or defining Q-sorts. In order to create the final factor estimate (Table 2), each Q-sort’s factor weight needs to be applied to its own item rankings (the PQMethod software automatically provides this information).

### Case study description

Kaiserslautern is a city in the south-west of Germany, located in the state of Rhineland-Palatinate on the French border. The city has 100,747 (2017) inhabitants. Forecasts indicate a total population loss of around 3% by 2020 (Stadt Kaiserslautern, 2016).

In the 19th century, the founding of numerous companies in the textile, metal and engineering sectors enabled Kaiserslautern to emerge as one of the most important industrial locations within the Palatinate. Industry flourished again after the Second World War (also due to the location of Opel), but the oil crises in 1973 as well as the land-intensive settlement structures of the military inhibited the further development of the industry. In the 1970s several companies went into crisis; in 1981, the Kammgarnspinnerei became insolvent, while Pfaff as well as Opel dismissed employees. The primary and secondary sectors declined in importance (11,133 employees in 2015), while the tertiary sector became a job engine (40,390 in 2015). Industrial foci are: the automotive supply industry, chemical industry, information technology and media, logistics, engineering, and utility vehicles.

The city of Kaiserslautern and the surrounding region face several challenges, despite the facts that they are the centre of the regional labour market and their gross domestic product (GDP) per inhabitant is above the German average. For years Kaiserslautern has had the highest per capita debt of all German cities and therewith a lack of investment capital. Furthermore the city and region are characterized by youth unemployment rates and low-skilled worker unemployment rates which are above the German average. Although various job opportunities have been created via active firm settlement policies as well as spin-off foundations of the HEIs, the skills requested do not match with those of the low-skilled workers who had previously been employed in industry.

It was in the late 1960s and early 1970s when the Technical University Kaiserslautern (TUK) as well as the University of Applied Sciences Kaiserslautern (UASK) were founded. The political intent was to realize a structural policy via HE policy. Both HEIs were founded with a clear expectation of spurring the economic development of the region and engaging with the regional environment. The initial focus was on natural sciences and technology, followed by the integration of social and economic sciences. Due to the foundation and attraction of cutting-edge research institutes (two Fraunhofer Institutes, the Max-Planck Institute as well as other research units) as well as international companies (e.g., John Deere) and the efforts of the UASK, the city was nominated ‘city of science’ in 2012.

## UNIVERSITIES’ REGIONAL ENGAGEMENT: BETWEEN TOP-DOWN INFLUENCE AND BOTTOM-UP MOTIVATION

This section presents the results of the Q-sort, followed by an analysis and discussion of the findings. The Q-sort methodology discloses two shared viewpoints in the case study Kaiserslautern. These two viewpoints differ in the importance they assign to organizational, regional and organizational-field-level influencing factors, according to the relative ranks they assign them. They are subsequently referred to as viewpoint 1 (factor 1) and viewpoint 2 (factor 2). The individual perspectives of stakeholders from regional development agencies (R), policy-makers (P), regional economy (W), as well as the HEIs (W = research, I = intermediary, V = administration) split between the two viewpoints.

Viewpoint 1 emphasizes the influential role of drivers from the universities’ organizational level (Table 3). It is characterized by the perceived importance of (1) the role of individual ‘institutional entrepreneurs’, (2) the need for third-party funds and public subsidies and funding programmes, and (3) HEI internal incentive systems (awards, reduction of teaching obligations, recognition of scientific achievements). HE policy and research policy of the national government and expectations towards regional engagement activities of universities which are published in the regional and national media as well as a change in the meaning of universities’ regional engagement are not perceived as playing an influential role.

**Table 3.** Ranking of factors influencing the occurrence of university–region interaction OF, factor from the organizational field; R, factor from the regional environment; U, factor from the university) due to their significance (3 = very influential, –3 = no influence).

Viewpoint 1	Viewpoint 2	Rank
Individual personalities (‘frontrunners’, ‘institutional entrepreneurs’) (U)	Change of meaning of HEI–region interactions in the national and international higher education system (OF)	3
Need for third-party funds (U)	Individual personalities (‘frontrunners’, ‘institutional entrepreneurs’) (U)	2
Public subsidies and funding programmes (OF)	The self-perception of the HEI (U)	2
HEI internal incentive systems (awards, reduction of teaching obligations, recognition for the scientific career) (U)	Economic policy and regional policy and their programmes and strategies (R)	1
Regional networks and governance structures (R)	Regional networks and governance structures (R)	1
The self-perception of the HEI (U)	Public subsidies and funding programmes (OF)	1
The regional absorptive capacity (R)	Competition with other HEIs (OF)	0
Economic policy and regional policy and their programmes and strategies (R)	The regional absorptive capacity (R)	0
The founding history as well as reason for the founding of the HEI (U)	The thematic focus of higher education policy and research policy of the national government (OF)	0
Prices and awards of the public sector (OF)	The founding history as well as reason for the founding of the HEI (U)	0
Competition with other HEIs (OF)	Prices and awards of the public sector (OF)	–1
The role model effect as well as experience of other HEIs with regional engagement (OF)	HEI internal incentive systems (awards, reduction of teaching obligations, recognition for the scientific career) (U)	–1
Higher education legislation and the therewith changes (e.g., evaluation criteria) (OF)	Need for third-party funds (U)	–1
The thematic focus of higher education policy and research policy of the national government (OF)	The role model effect as well as experience of other HEIs with regional engagement (OF)	–2
Expectations towards regional engagement activities of universities that are published in the regional and national media (R)	Higher education legislation and the therewith changes (e.g., evaluation criteria) (OF)	–2
Change of meaning of HEI–region interactions in the national and international higher education system (OF)	Expectations towards regional engagement activities of universities which are published in the regional and national media (R)	–3

Note: HEI, higher education institution.

Viewpoint 2, on the other hand, emphasizes the influence of factors from the organizational-field-level as well as intra-organizational factors. (1) The change of meaning of universities’ regional engagement on the national and international level ranks first in terms of perceived importance, followed by (2) the self-perception of the university, and (3) the role of individual personalities. Nearly no influence is attributed to the role model effect, experiences of other HEIs with regional engagement activities, or HE legislation. Expectations towards regional engagement activities of universities which are published in the regional and national media are seen as exerting no influence at all.

Surprisingly, the regional environment and drivers therein, such as regional networks and governance structures, economic policy and regional policy, as well as the regional absorptive capacity seem to be of low to no importance with regards their influence on the occurrence of HEI–region collaborations.

The Q-factor analysis provides an objective account of commonalities between subjective viewpoint, in this case of what matters in influencing engagement in the Kaiserslautern region. To understand these results in the context of the Kaiserslautern region, it is necessary to take into account other empirical data gathered within the case study.

One of the emerging perspectives relates to a highly institutionalized reading of regional engagement, and can be traced to the strong policy push to create universities as drivers of regional development in the Palatinate. The second perspective is a much more diverse view, and reflects the fact that universities engage in many kinds of engagement activities beyond this narrow institutional engagement, driven through individual interaction with regional partners. More information is provided on these two perspectives below.

### Viewpoint 1: Institutionalized economic understanding of regional engagement

Viewpoint 1 is shaped by the influence of high personal motivation, the need for third-party funds, as well as organizational support and incentives. The underlying understanding of regional engagement is an economic one, insofar as knowledge transfer to regional businesses and industry, research and development (R&D) cooperation and spin-off creations receive foremost attention.

Although the importance of the founding history is not consciously outlined as influential (array 0), viewpoint 1 is historically imprinted. The Minister for Culture and Education of Rhineland-Palatinate argued in the 1970s for the establishment of a two-campus university in Trier and Kaiserslautern, with Kaiserslautern having a more technical profile. The education-policy driven motivations were (1) the continuing shortage of teachers in the federal state, (2) the increasing number of students with an university entrance qualification, and (3) the lack of study places due to the *numerus clausus* system. While at the location in Trier the focus was on social sciences and humanities, the location in Kaiserslautern was clearly aimed at providing ‘development aid’ to the region, which was also mentioned as a reason to focus on natural and technological sciences. The TUK started its operation in 1969 with the faculties of mathematics, physics and technology as well as environmental and spatial planning, biology and chemistry, plus engineering a year later.

Even at a time when there was no encouragement for engagement, engagement still happened. The understanding of regional engagement at the TUK was for decades strongly shaped by the personal engagement by two professors in physics and mathematics. Although it was rather uncommon and referred to as ‘unscientific’ at that time to approach partners in the regional economy and offer solutions to their everyday challenges, these professors perceived it as their normative obligation as well as an opportunity to raise additional funding. Especially the mathematics professor was well known for approaching regional industries and companies, offering them solutions for their everyday problems, and establishing cooperation. Via student projects, informal consulting and contract research solutions for the problems of regional businesses and industry partners have been elaborated, which later on developed into more formal R&D cooperation. It was also this professor who established the first Fraunhofer Institute on the campus of the university and initiated the ‘Science Alliance’ in 2007, a network between university researchers, university management, the UASK and partners from the regional economy.

Based on the increasing number of regional engagement activities, the university management established a TTO to institutionalize and foster university–industry cooperation in other areas as well as to support spin-off foundations. Furthermore, incentives such as a reduction of teaching obligations for engagement in industry cooperation are established at both HEIs. These types of regional engagement activities became well known for the TUK as well as the UASK, although they are limited to the natural science and technology faculties. These economically focused activities benefit from new organizational units, reduction of teaching obligations, recognition of scientific achievements, and the mission and self-perception of the HEIs.

### Viewpoint 2: Regional engagement as a multifaceted activity with a social mission

Viewpoint 2 in the case of Kaiserslautern describes regional engagement activities in the broader context of an overall normative and cognitive change towards a more comprehensive understanding of universities’ regional engagement in the German HE system. Although the HE system is the responsibility of the federal states, the German government exerts influence via flagship reports (e.g., role of universities in sustainability transitions, German Advisory Council on Global Change: WBGU, 2011) or funding programmes (e.g., the programme ‘Innovative HEI’ to support third-mission activities). Furthermore, most federal ministries foster the implementation of boards of trustees (‘Hochschulkuratorium’) within the HEIs. They represent new organizational units which, in Rhineland-Palatine, assemble 13 external representatives from the regional and federal level (three representative from the ministry, three representatives elected by the federal parliament and seven regional representatives elected by the university).

The main purpose of this board of trustees is to consult with the university senate on how to better anchor the HEI in the region and regional knowledge demands, as well as (regional) need for further education opportunities. Although this top-down policy push is aimed towards strengthening the regional mission of the respective HEI, the focus of the board of trustees depends very much on the elected representatives. Interview partners who support viewpoint 2 emphasized that apart from the commercialization-based activities of TUK and UASK there are several engagement activities which are undertaken by faculty from social and economic sciences. These activities entail consultation for public authorities (especially the city government), participation in regional initiatives and platforms as well as student projects to support the city government in topics such as integration of the long-term unemployed and other forms of segregation. Most of these activities are accompanied by small project budgets if not performed on a completely voluntary basis, which is also reflected in the low ranking of the driver ‘need for third-party funds (U)’.

The respective interview partners point out that these kind of regional engagement activities are often seen in a field of tension between provincialism and the need to compete for international excellence. University institutes engaged in these activities do not publish as much or raise as much third-party funding as others do, which brings them into the situation of having to justify these activities. Due to these aspects it is noted that these kinds of regional engagement activities are conducted mainly by senior personnel with mostly permanent working contracts, who feel a normative obligation due to the role/position they occupy. Though these types of engagement activities are tolerated, they are not perceived as being actively supported or rewarded within the organization (low rank of the driver ‘HEI internal incentives’: –1).

Based on these two viewpoints and the associated reflections, one can deduce a couple of ‘stylized facts’. First, individual personalities play an outstanding role in pursuing regional engagement based on their convictions, values, norms and motivation. Second, networks are important but not dominant in the subjective deliberation process towards regional engagement. Third, technology transfer is anchored in university strategies and institutional support (via infrastructure, service, and personnel). In contrast, this is not the case for non-technological knowledge exchange, which is more reliant on individuals. Fourth, the region’s absorptive capacity does not decisively shape engagement behaviours and collaborative structures.

### Regional deliberation processes in Kaiserslautern

The investigations confirm that the decision to engage regionally is a deliberation process which is simultaneously influenced by several drivers operating on multiple levels. This paper corroborates the essential role of individual personalities (‘frontrunners’, ‘institutional entrepreneurs’) for initiating regional engagement activities and serving as role models. They undertake commercialization- or non-commercialization-focused regional engagement activities due to their normative conviction of their role (e.g., to give back to society), a feeling of responsibility (e.g., to ensure financing), or also the need to fulfil certain evaluation criteria (e.g., amount of third-party funds raised). Interestingly, within the present case study the role of regional networks and governance structures ranks somehow in a neutral position. This is surprising as there are strong networks between the university and various regional stakeholders. One tentative explanation is that they are taken for granted and thus not consciously given the attention they might deserve. Especially new organizational structures such as boards of trustees at HEIs, which was initiated by the ministry, but also platforms such as the ‘Science Alliance’ foster and strengthen the relational ‘thickness’ in the region. The latter was bottom-up initiated by one professor of the TUK, who became an agenda-setter in shaping regional networks. What becomes evident is that those activities which refer to the linear transfer and commercialization of academic knowledge (R&D cooperation, spin-off foundations, etc.) enjoy a high degree of support and institutionalization by the university management as well as ministry, while non-commercial regional engagement activities rely very much on the ambitions of individual personalities inside and outside the HEI.

In contrast to the literature (e.g., Arbo & Benneworth, 2007; Fürst & Back, 2011), the absorptive capacity of the region is not emphasized as an important influencing factor on the occurrence of HEI–region collaborations. Reasons for this can be found in the fact that for non-commercial regional engagement activities public authorities and regional development agencies are most often the recipients, while small and medium-sized enterprises (SMEs) are also relevant in the frame of teaching activities. With regards commercialization based activities, the university shapes the regional absorptive capacity via the foundation of spin-offs, which most often located nearby the university and consequently become partners in R&D cooperation. These new business foundations are often the first working places for graduates and thereby compensate the lack of high-tech companies in the region.

Overall the impression arises that the TUK as well as the UASK decide about their regional engagement activities and focus autonomously. They do not really feel the pressure to legitimize their existence as their contribution to the development as well as the image of the region is unquestioned. As there are only two HEIs in the region, which cooperate more often than they compete (Uyarra, 2010), there is a lack of pressure from this side.

## CONCLUSIONS

Taking the example of one single case study investigation the region of Kaiserslautern (D), the paper shows how the multilevel environment, within which the university is located, influences the universities’ regional engagement. It becomes apparent that the occurrence of regional engagement activities (commercial and non-commercial) cannot be explained by individual indicators (e.g., scientific field, academic age) but has to be viewed more systemically.

The identified two viewpoints illustrate that university engagement is a balancing act between individual autonomy and expectations expressed by the university environment. Two shared viewpoints were identified among stakeholders, each of which is shaped by different intra-organizational, regional and organizational-field factors. One perspective relates to a highly institutionalized reading of regional engagement, and can be traced to the strong policy push to create universities as drivers of regional development in the Palatinate. The second perspective is a much more diverse view, and reflects the fact that universities are engaged in many kinds of engagement activities beyond this narrow institutional engagement, driven through individual interaction with regional partners.

The case study and analysis highlighted four stylized facts evident in this multilevel perspective. The first and arguably most influential is the role of individual personalities (‘frontrunners’). These frontrunners are well known in the region and important contact persons for regional stakeholders. Furthermore they function as role models for their university’ colleagues. Second, networks are important but not dominant in the subjective deliberation process towards regional engagement. Third, technology transfer is anchored in university strategies and institutional support (via infrastructure, service, personnel). Non-technological knowledge exchange, on the other hand, is reliant on individuals.

The study also sheds a light on the persistence of a number of multilevel tensions. On the one hand, universities and their members have a rather high autonomy in defining their understanding of regionalism. On the other hand, as an open system they are prone to regional expectations and general developments of the (inter-)national HE system, which affect the subjective deliberation process and have to be considered in the process of creating a strategy. A strategy which is based on the pipeline-dominated approach covers only a small part of universities’ regional engagement activities and gives an incomplete representation of the wider processes of knowledge exchange and co-production as well as networking and collaboration between university members and regional stakeholders. Recognizing and incorporating the subjective deliberative processes, which are influenced by the multilevel environment the university is located in, may lead to a more systematic and balanced perception of the role an HEI can play within its region.

## NOTE

1 Significant factor loading is calculated as follows: 2.58*(1/√number of items in the Q-set).
